# Clinicopathological characteristics of gastric cancer patients with dermatomyositis and analysis of perioperative management: a case series study

**DOI:** 10.3389/fsurg.2023.1276575

**Published:** 2023-11-01

**Authors:** Muerzhate Aimaiti, Haoyu Zhang, Dilidaer Aikebaier, Bo Ni, Hanlin Yin, Zhongyi Dong, Yeqi Zhang, Yujing Guan, Long Bai, Shuchang Wang, Xiang Xia, Zizhen Zhang

**Affiliations:** ^1^ Department of Gastrointestinal Surgery, Renji Hospital, School of Medicine, Shanghai Jiao Tong University, Shanghai, China; ^2^ Department of General Medicine, Kashe District Second People’s Hospital, Xinjiang, China; ^3^ Department of Rheumatology, Renji Hospital, School of Medicine, Shanghai Jiao Tong University, Shanghai, China

**Keywords:** dermatomyositis, gastric cancer, clinicopathological characteristics, postoperative complication, perioperative management

## Abstract

**Background:**

This study aimed to investigate the clinical characteristics of gastric cancer (GC) patients with dermatomyositis (DM) and summarize the perioperative outcomes.

**Methods:**

The clinical and pathological data of five patients diagnosed with co-occurring DM and GC (DM-GC group) were retrospectively analyzed, who were admitted to the Department of Gastrointestinal Surgery at Ren ji Hospital, Shanghai Jiao Tong University, between January 2012 and April 2023. Their data were compared with 618 GC patients (GC-1 group) from September 2016 to August 2017 and 35 GC patients who were meticulously screened from 14,580 GC cases from January 2012 and April 2023. The matching criteria included identical gender, age, tumor location, TNM stage, and surgical procedure (7 GC patients were matched for each DM-GC patient).

**Results:**

Analysis indicated that the DM-GC group comprised four female and one male patient. The female proportion was significantly higher (*P* = 0.032) than that of GC-1 group. In DM-GC group, four DM patients were diagnosed as GC within 12 months. One DM patients was diagnosed as GC within 15 months. Among them, four patients presented with varying degrees of skin rashes, muscle weakness while one patient had elevated CK levels as the typical symptom. Similarly, the preoperative tumor markers (CA-199 and CA-125) in the DM-GC group were significantly higher than normal levels (CA-199: 100 vs. 28.6%, *P* = 0.002; CA-125: 40 vs. 2.9%, *P* = 0.003) compared to GC-2 group. Moreover, postoperative complication incidence and the length of hospital stay were significantly higher in the DM-GC than GC-2 group [complication rate: 40 vs. 8.6%, *P* = 0.047; hospital stay: 15 days (range: 9–28) vs. 9 days (range: 8–10), *P* = 0.021].

**Conclusion:**

GC Patients with dermatomyositis are more prone to experience postoperative complications and longer hospital stay.

## 1. Introduction

Gastric cancer (GC) is a malignant tumor originated from the epithelial cells of the stomach and is ranked the fifth most common cancer globally and the fourth leading cause of cancer-related deaths globally (1–3). The exact etiology of GC remains unclear; however, it has been associated with various factors such as geographic and environmental influences, dietary and lifestyle choices, *helicobacter pylori* infection and genetic predisposition (approximately 10% of GC cases exhibit familial clustering) (4). Recent epidemiological trends suggested a potential reversal in the incidence of GC and the dominance of female cases were observed over the past few decades, with leading contributing factors (*helicobacter pylori* infections) and an increased prevalence of autoimmune diseases. Consequently, the early identification of high-risk GC individuals, particularly those with autoimmune disorders, is vital for achieving early diagnosis and reducing GC-related mortality rates (5, 6).

Dermatomyositis (DM) is an autoimmune disease characterized by idiopathic inflammatory myopathy (IIM) with involvement of the skin and skeletal muscles, which could potentially affect other systems like the lungs, digestive tract, heart, and kidneys, and in some cases, DM patients may even develop cancers (7). Although most DM cases lack a clear etiology, a study by Spanish researchers in 2018 proposed a possible association of mutations in the transcriptional intermediary factor 1 (TIF1) (8). The pathogenesis DM is believed to involve the dysregulated immune system, cross-immune reactions, viral infections, and other genetic factors. A possible association between DM and GC was first reported in 1976, which attracted the attention of researchers for investigating DM and tumor relationships (9). The prevalence rate of cancers in DM patients had been reported to be significantly higher than in the general population tumors in DM patients had been reported to be significantly higher than in the general population, with a 5-year survival rate ranging from 10% to 56%, which is much lower compared to 60% to 90% observed in DM patients without malignancies, thereby making malignant tumors a severe complication affecting DM patients' prognosis (10).

Considering numerous autoimmune disease cases and gastrointestinal malignancies encountered in clinical practice, conducting in-depth investigations for potential associations between DM and GC has become imperative. Therefore, this study aimed to comprehensively analyze the clinical and pathological characteristics of patients presenting with DM with GC to provide valuable clinical insight, which is envisaged to significantly contribute to more effective perioperative management strategies for DM-GC patients.

## 2. Data and methods

### 2.1. Study subjects

A retrospective analysis of clinical data from five GC patient with DM admitted to Department of Gastrointestinal Surgery, Renji Hospital, School of Medicine, Shanghai Jiao Tong University, between November 2012 and April 2023 (DM-GC group). Their clinical data was compared to 618 GC patients between September 2016 and 2017 (GC-1 group). Additionally, a control group (GC-2) was also established by carefully matching 35 patients from a total of 14,580 GC cases from same hospital between January 2012 and April 2023, ensuring their gender, age, tumor location, and TNM stage were identical to those of the five patients in the DM-GC group (7 GC patients were matched for each DM-GC patient).

The five dermatomyositis patients underwent routine oncology screening according to the treatment guidelines for dermatomyositis at the time of their initial diagnosis. Specific symptoms (as Stomachache, Haematemesis, Emesis, weight loss) related to gastric cancer occurred during the treatment of dermatomyositis and post-treatment follow-up, leading to the discovery of gastric cancer during a review of tumor-related examinations.

### 2.2. Research methods

#### 2.2.1. Data collection

The data of patients were collected, including general information like gender, age, body mass index (BMI), preoperative immunohistochemistry results, preoperative tumor markers (CA-199, CA-125), creatine kinase (CK) levels, postoperative hospital stay, postoperative complications, including tumor location, tumor size, surgical time, intraoperative blood loss, surgical procedure, digestive tract reconstruction and number of retrieved lymph nodes. Patients were carefully matched from a database of 14,580 GC cases admitted in the same hospital between January 2012 and April 2023 regarding gender, age, tumor location, and TNM stage with five DM-GC group patients. The postoperative complications based on the Clavien-Dindo classification system were also evaluated. GC staging was followed by American Joint Committee on Cancer (AJCC) staging criteria (11, 12).

Postoperative follow-up was conducted through various means such as phone calls, WeChat, or outpatient reviews, including evaluations of symptoms, physical and hematological examinations, imaging studies, gastroscopy, and other relevant assessments.

#### 2.2.3. Statistical analysis

The data were analyzed using SPSS (version 22.0) software. Measurement data were compared between the two groups using t-tests for data with a normal distribution, while rates were compared using chi-square tests.

## 3. Results

### 3.1. General characteristics

The data analysis showed that the DM-GC group (Table 1) consisted of four female and one male patient, with a median age of 69 years (ranging from 49 to 83), Out of the patients, four were aged 60 years or above, while one was below the age of 60, as is shown in Table 1.

**Table 1:** Clinicopathological characteristics of 5 patients with dermatomyositis combined with gastric cancer.

Clinicopathological characteristics	Case 1	Case 2	Case 3	Case 4	Case 5
Age	83	69	73	66	49
Gender	Female	Female	Male	Female	Female
Tumor location	Distal segment	Distal segment	Proximal segment	Distal segment	Middle segment
T stage	T4	T4	T4	T4	T4
N stage	N2	N0	N3	N1	N3
TNM stage	Stage III	Stage II	Stage III	Stage III	Stage III
Blood vessel invasion	Yes	Yes	Yes	Yes	Yes
Nerve invasion	Yes	Yes	Yes	Yes	Yes
Surgical procedure	Distal gastrectomy	Distal gastrectomy	Total gastrectomy	Distal gastrectomy	Total gastrectomy
Digestive tract reconstruction	Billroth—II	Billroth—II	Roux—en—Y	Billroth—I	Billroth—I
CEA	Elevated	Normal	Elevated	Normal	Elevated
CA-199	Elevated	Elevated	Elevated	Elevated	Elevated
CA-125	Elevated	Normal	Normal	Normal	Elevated
Postoperative complications	Yes	No	No	No	Yes
Postoperative hospital stay (Days)	32	15	9	9	24
Dermatomyositis related characteristics					
First symptoms	CK value increase	Exanthema Hypokinesia	Exanthema Hypokinesia	Exanthema Hypokinesia	Exanthema Hypokinesia
Symptoms at GC diagnosis	Haematemesis	Stomachache	Stomachache, Emesis	Stomachache weight loss	Stomachache
GC diagnostic method	AbdomenCTA Gastroscope	Gastroscope	PET-CT	AbdomenCTA Gastroscope	AbdomenCTA Gastroscope，PET-CT
Interval between dermatomyositis and subsequent gastric cancer diagnosis (months)	15	11	4	7	3
Preoperative creatine kinase (CK)	2,423	890	3,133	37	87
Postoperation creatine kinase (CK)	40	425	157	19	20
Myositis-specific antibodies (MSA)	Anti-TIF1-*γ*	Undetected	Undetected	Anti-TIF1-γ	Anti-TIF1-γ
Exanthema	No	Yes	Yes	Yes	Yes
Heliotrope	No	Yes	No	Yes	No
Gottron	No	Yes	Yes	Yes	Yes
Hypokinesia	No	Yes	Yes	Yes	Yes
Manual muscle testing score (MMT)	5	4	4	4	4
Interstitial pneumonia	No	No	No	No	No
Deglutition disorders	No	No	No	No	No
Corticosteroid volume (mg/day)	40	40	60	40	30
Immunosuppressive drugs	Yes	Yes	No	No	Yes
Immunosuppressive drugs types	MTX AZA	Tac	No	No	Tac

MTX, methotrexate; AZA, azathioprine; Tac, tacrolimus.

In all of the selected cases, the diagnosis of DM preceded the diagnosis of GC. As part of the treatment guidelines for dermatomyositis at the time of their initial diagnosis, the five dermatomyositis patients underwent routine oncology screening. During the course of dermatomyositis treatment and post-treatment follow-up, specific symptoms related to gastric cancer emerged, prompting a review of tumor-related examinations. This review led to the detection of gastric cancer within a time span ranging from 3 to 15 months. Four patients presented varying degrees of skin rashes, muscle weakness as initial DM symptoms, while one exhibited elevated CK level as an initial symptom. All patients in the DM-GC group showed significantly elevated levels of preoperative CA-199, and two patients showed significantly higher CA-125 levels (normal range: 0–35 U/ml). Regarding tumor location, one case had a tumor in the proximal, one in the middle, and three in the distal segments. In compliance with the principles of GC treatment, all five patients underwent radical GC surgery in our hospital, including four open surgeries and one laparoscopic surgery. Furthermore, two patients underwent total gastrectomy, while three underwent distal gastrectomy. Postoperative pathological examination revealed that the tumor stages in the DM-GC group were predominantly advanced, with one case classified as stage II and four as stage III. Postoperative pathological examinations revealed that all five patients exhibited blood vessels and nerve invasion. The average postoperative hospital stay was 17.8 days (9–32) (Table 1).

### 3.2. Comparison of clinical and perioperative data before matching

Comparing the data of patients in the DM-GC group with 618 GC patients (GC-1 group) revealed that the proportion of female patients in the DM-GC group was significantly higher than the GC-1 group (80% vs*.* 34.1%, *P* = 0.032). However, no significant differences were observed between the two groups regarding age, tumor location, clinical stage, postoperative complications, and postoperative hospital stay (*P* > 0.05) (Table 2).

**Table 2:** Comparison of clinicopathological characteristics between dermatomyositis with gastric cancer (DM-GC group) and gastric cancer (GC-1 group) patients before matching.

Clinicopathological characteristics	DM-GC group (*N* = 5)	GC-1 group (*N* = 618)	*P* value
Age			0.446
≤60 years	1 (20.0)	225 (36.5)	
>60 years	4 (80.0)	393 (63.5)	
Gender			0.032
Male	1 (20.0)	407 (65.7)	
Female	4 (80.0)	211 (34.1)	
Tumor location			0.735
Proximal	1 (20.0)	134 (21.7)	
Middle	1 (20.0)	214 (34.6)	
Distal	3 (60.0)	270 (43.7)	
T stage			0.334
T1	0 (0.0)	144 (23.3)	
T2	0 (0.0)	66 (10.7)	
T3	0 (0.0)	41 (6.6)	
T4	5 (100.0)	367 (59.4)	
N stage			0.777
N0	1 (20.0)	253 (41.0)	
N1	1 (20.0)	88 (14.2)	
N2	1 (20.0)	123 (19.9)	
N3	2 (40.0)	154 (24.9)	
M stage			0.536
M0	5 (100.0)	574 (92.9)	
M1	0 (0.0)	44 (7.1)	
TNM stage			0.357
Stage I	0 (0.0)	180 (29.1)	
Stage II	1 (20.0)	111 (18.0)	
Stage III	4 (80.0)	282 (45.6)	
Stage IV	0 (0.0)	45 (7.3)	
Postoperative complications			0.301
Yes	2 (40.0)	130 (21.0)	
No	3 (60.0)	488 (79.0)	
Postoperative hospital stay (days)	15 (9∼28)	10 (9∼11)	0.119

### 3.3. Comparison of clinical and pathological characteristics after matching

After matching, a control group (GC-2) comprising 35 patients were selected from 14,580 GC patients admitted to Renji Hospital, Shanghai Jiao Tong University School of Medicine, from January 2012 to April 2023. Matching variables included age, gender, tumor location, pT stage, pN stage, pM stage, clinical stage, and surgical procedure (Table 3). Comparative assessment of DM-GC and GC-2 group revealed significant differences (*P* < 0.05) in preoperative tumor marker CA-199 and CA-125 levels (CA-199: 100% vs. 28.6%, *P* = 0.002; CA-125: 40% vs. 2.9%, *P* = 0.003), postoperative hospital stay, and postoperative complication rates. Similarly, the incidence of postoperative complications was significantly higher in the DM-GC group compared to the GC-2 group (40% vs*.* 8.6%, *P* < 0.05), with significantly longer postoperative stay (DM-GC = 15 days [range: 9–28] vs. GC-2 = 9 days [range: 7–10], *P* < 0.05) (Table 3).

**Table 3:** Comparison of clinicopathological characteristics characters between dermatomyositis with gastric cancer (DM-GC group) and gastric cancer (GC-2 group) patients after matching.

Clinicopathological characteristics	DM-GC (*N* = 5)	GC-2 (*N* = 35)	*P* value
Age [*n* %]			1.000
≤60 years	1 (20.0)	7 (20.0)	
>60 years	4 (80.0)	28 (80.0)	
Gender [*n* %]			1.000
Male	1 (20.0)	7 (20.0)	
Female	2 (80.0)	28 (80.0)	
Tumor location [*n* %]			1.000
Proximal	1 (20.0)	7 (20.0)	
Middle	1 (20.0)	7 (20.0)	
Distal	3 (60.0)	21 (60.0)	
T stage [*n* %]			1.000
T1	0 (0.0)	0 (0.0)	
T2	0 (0.0)	0 (0.0)	
T3	0 (0.0)	0 (0.0)	
T4	5 (100.0)	35 (100.0)	
N stage [*n* %]			1.000
N0	1 (20.0)	7 (20.0)	
N1	1 (20.0)	7 (20.0)	
N2	1 (20.0)	7 (20.0)	
N3	2 (40.0)	14 (40.0)	
TNM stage [*n* %]			1.000
Stage I	0 (0.0)	0 (0.0)	
Stage II	1 (20.0)	7 (20.0)	
Stage III	4 (80.0)	28 (80.0)	
Stage IV	0 (0.0)	0 (0.0)	
Surgical procedure [*n* %]			1.000
Distal gastrectomy	3 (60.0)	21 (60.0)	
Total gastrectomy	2 (40.0)	14 (40.0)	
Postoperative complications [*n* %]			0.047
Yes	2 (40.0)	3 (8.6)	
No	3 (60.0)	32 (91.4)	
Postoperative hospital stay (days)	15 (9∼28)	9 (7∼10)	0.021
CEA [*n* %]			0.297
Normal	2 (40.0)	26 (74.3)	
Elevated	3 (60.0)	9 (25.7)	
CA-199 [*n* %]			0.010
Normal	0 (0.0)	25 (71.4)	
Elevated	5 (100.0)	10 (28.6)	
CA-125 [*n* %]			0.041
Normal	3 (60.0)	34 (97.1)	
Elevated	2 (40.0)	1 (2.9)	

## 4. Discussion

Dermatomyositis (DM) is an autoimmune disease characterized by idiopathic inflammatory myopathy primarily affecting the skin and skeletal muscles. It predominantly affects middle-aged females, and DM patients exhibit a significantly higher risk of developing malignancies compared to the general population. Studies have shown that the incidence of malignancy in adult dermatomyositis patients is approximately 4.66 times higher than that in the general population (13). The estimated occurrence of malignancy in DM patients ranges from 13% to 43%, with gastrointestinal and ovarian cancers being the most common malignancies observed (14, 15). Malignancy has emerged as one of the leading causes of mortality in DM patients, and its onset is typically observed above the age of 40, with an increasing risk of concurrent malignancies with advancing age (16, 17). Notably, females have a threefold higher risk of developing malignancies than males in the context of DM (18, 19). Several studies have identified an age of onset over 40 years as an independent risk factor for the association between DM and malignancy (16). The average age of initial DM diagnosis in patients with malignancy is significantly higher than that in patients without malignancy (68.8 years vs. 52.4 years), and DM patients aged 52 years or older are more susceptible to developing malignancies (20). Consistent with previous reports, our study observed a median age of 68 years (ranging from 49 to 83 years) for DM patients with concurrent gastric cancer, and a male-to-female ratio of approximately 4:1, aligning with the prevailing trends in the literature.

Malignancies can be diagnosed before, simultaneously with, or after the DM diagnosis, whereas in most cases, malignancies are detected after the diagnosis of DM, with the highest incidence occurring within the first 1 years (21). The risk of malignancy development is particularly elevated within the first year after DM diagnosis, gradually declining in the subsequent years but remaining high than that in general population (17, 22). Comprehensive tumor screening is pivotal within the first year after DM diagnosis, with dynamic and systematic screening continuing for up to 3 years post-diagnosis (18). In China, most (74%–81.8%) of malignancies in DM patients are identified after the DM diagnosis, with most patients initially seeking treatment in dermatology or rheumatology departments (23). In our study, all five patients in the DM-GC received routine oncology screening for dermatomyositis and malignant tumors were excluded according to the treatment guidelines for dermatomyositis at the time of initial diagnosis. Specific symptoms related to gastric cancer occurred during the treatment of dermatomyositis and post-treatment follow-up, then they were referred to the gastrointestinal surgery department. Importantly, all patients were diagnosed with GC within 1 years after their DM diagnosis, with an interval ranging from 3 months to 15 months, reinforcing the significance of vigilant tumor screening for gastric malignancy in DM patients during the early post-diagnosis period.

Due to the significant risk of malignancy in DM patients and its substantial impact on mortality, timely screening for malignancies in DM patients with high-risk factors is vital. Effective tumor screening tools are essential for early diagnosis. Screening for malignant tumors in patients with dermatomyositis should be tailored by considering various factors, including age, symptoms, MSA (Myositis-Specific Antibodies), and others, to accurately assess the risk of malignancies. screening methods encompass a comprehensive array of evaluations, including physical examinations, blood tests, urine and stool analyses, and tumor markers (CA-125, CA19-9 for males, and PSA for males depending on age). Imaging examinations such as mammography, pelvic ultrasound, chest, abdominal, and pelvic computed tomography (CT)/MRI, positron emission tomography (PET)-CT, cytology (smear of exudated cervical cells), nasopharyngoscopy, and gastroscope are also employed as part of our screening protocol. Female patients undergo specific screening for gynecological and breast cancers. For patients diagnosed with DM, should be screened for malignant tumors for at least 3 years. Patients who are positive for anti-TIF1-γ antibodies and anti-NXP-2 antibodies should undergo screening for at least 5 years (17, 24).

The novel autoantibody anti-p155 antibody (anti-TIF1-γ antibody) has recently emerged as a valuable diagnostic tool, whose presence is closely associated with cancer incidence in adult patients, with an estimated rate of 22%–100% of anti-TIF1-γ antibody-positive patients diagnosed with cancer (25). A systematic retrospective analysis of 327 patients demonstrated that DM patients positive for anti-TIF1-γ antibodies had a 27 times higher rates of concurrent malignancy than those in negative group (26).

A study involving 102 DM patients indicated that within the first year after DM diagnosis, the risk of developing malignancies was highest when tumor markers CA-125 and CA-199 showed elevated levels, with persistent elevated CA-125 levels mounting the risk of concurrent malignancies. The assessment of CA-125 and CA-199 could be helpful biomarkers for assessing the risk of malignancies in patients with DM and polymyositis, thus warranting their inclusion in cancer research (27). In this study, the DM-GC group showed significantly higher levels of CA-199 and CA-125 before surgery compared to the GC-2 group (CA-199: 100% vs. 28.6%, *P* = 0.002; CA-125: 40% vs. 2.9%, *P* = 0.003) (Table 3).

Formal guidelines for high-risk screening in DM patients with concurrent GC must be improved. Conventional imaging tools have limitations in detecting early malignancies, necessitating exploring more effective screening methods. As an increased malignancy risk is present for ≤5 years after DM onset, some authorities recommend annual imaging until that time point is reached (24). Thus, combined anti-TIF1-γ antibody testing with imaging examination are advocated for DM patients. Positive antibody patients should undergo imaging examination annually for 3–5 years, while negative antibody patients may only need imaging examination at the time of diagnosis.

According to tumor treatment principles is essential in treating DM patients with concurrent malignancies. Early detection of malignancies is critical, and prompt surgical resection or other appropriate treatments should be pursued if surgical indications are presented; for cases where surgery was not indicated, radiotherapy and chemotherapy should be considered as initial treatments as the followed adjuvant therapy based on postoperative pathology and malignancy staging. The primary goal of treating DM patients with concurrent malignancies is to diagnose and address the tumor while improving muscle and skin symptoms. Consequently, multidisciplinary consultations are required to assess surgical and anesthesia risks. The extent of DM lesions should be evaluated, especially if the abdominal skin is involved, as this may impact surgical considerations.

Moreover, the impact of DM on essential functional muscles, such as respiratory and pharyngeal muscles, should be assessed to optimize anesthesia and recovery outcomes. Surgery should be postponed in cases where DM affects these muscles until respiratory and pharyngeal muscle symptoms are improved. Notably, successful treatment of the underlying malignancy may lead to improvements or complete resolution of DM symptoms (19, 28). In this study, 3 of the 5 patients with dermatomyositis had abnormal CK values, and the CK values of the 3 patients with gastric cancer decreased significantly after surgery, and the symptoms of skin rash and weakened dermatomyositis have improved.

The primary medications used in dermatomyositis (DM) are corticosteroids and immunosuppressants (29). Systemic application of corticosteroids is the first choice for treating dermatomyositis, which can be divided into three stages: initial stage, reduction stage, and maintenance stage. Initial stage: The corticosteroid dose is generally 0.75–1 mg/kg prednisone (maximum 80 mg/day). Reduction stage: Most patients show significant improvement after 4–6 weeks of medication, mainly manifested by a significant decrease in muscle enzymes and a significant recovery of muscle strength. At this time, the reduction stage can be entered. Corticosteroid reduction at this stage should be based on the specific condition of the patient, such as the comprehensive consideration of muscle enzyme decline and muscle strength recovery.

Maintenance stage: After the corticosteroid is reduced to the lowest maintenance dose, it is usually maintained for 2 years or more to reduce recurrence (30, 31). A single corticosteroid is only suitable for some mild dermatomyositis patients; most patients need to use immunosuppressants. Immunosuppressants are usually added before and after the initiation of corticosteroid reduction to achieve early corticosteroid reduction, reduce the cumulative dosage of corticosteroid, and reduce the recurrence rate (31, 32). However, glucocorticoids may cause specific side effects during surgery, such as gastric and intestinal ulcers, gastrointestinal bleeding and perforation, impaired wound healing, disturbances in fat and electrolyte metabolism, and osteoporosis. Yet, the occurrence of most of these side effects depends on the dosage and duration of treatment (33).

Short-term glucocorticoid use can promote wound healing for patients undergoing substantial GC resection and is devoid of significantly increased glucocorticoid-related side effects. In this study, one DM patient with concurrent GC had a history of long-term high-dose oral glucocorticoid use (methylprednisolone succinate) and experienced an anastomotic leak after surgery. Whether perioperative glucocorticoids should be used in patients with DM and concurrent GC requires consultation between dermatologists and rheumatologists, comprehensive understanding of the patient's condition, strict adherence to indications and contraindications for glucocorticoid use, proper dosing, withdrawal methods, as well as close patient observation, to minimize the side effects and complications.

The prognosis of DM patients with concurrent malignancies is generally unfavorable, often characterized by widespread tumor metastasis, secondary infections, and systemic failure, which remain the primary cause to mortality. Overall, the survival rate of DM patients with concurrent malignancies is lower than those with other comorbidities, with a worse prognosis, particularly in cases with higher malignancy levels. Hence, early detection of malignancies is of utmost importance, and prompt initiation of surgical resection or appropriate treatments is critical for DM patients with tumor-related conditions (34, 35).

In our study, all five cases of DM with concurrent GC were found to be in advanced stages, with all of cases classified as pT4. Additionally, all DM-GC patients had nerve and vascular invasion. Following surgery, two patients experienced postoperative complications, one with a gastric paralysis and another with an anastomotic leak. The DM-GC group displayed significantly higher rates of postoperative complications and more extended hospital stays when compared to the GC-2 group. Moreover, the proportion of postoperative complications was significantly (*P* < 0.05) higher in the DM-GC group than in the GC-2 group (40% vs. 9.1%, *P* = 0.047), as well as significantly prolonged hospital stay (15 days, range: 9–28 days vs*.* 9 days, range: 8–10 days, *P* = 0.021).

It is worth noting that while previous studies have explored this association through case reports and meta-analyses, or have focused on the broader connection between autoimmune diseases and gastric cancer or dermatomyositis and malignancies, few have conducted a dedicated analysis of the relationship between GC and DM in isolation. This distinction underscores the novelty of our study (5, 36–38). To ensure the reliability of our findings, we meticulously crafted our control group (GC-2 group) by employing strict matching criteria. Specifically, we identified suitable control subjects from a pool of 14,580 GC patients. These criteria encompassed gender, age, tumor location, TNM stage, and surgical procedure, mirroring the characteristics of the patients in the DM-GC group. This rigorous matching process bolsters the robustness of our comparative analysis. The paucity of literature addressing perioperative complications in DM patients with GC is a notable gap in the field. We believe that our article significantly contributes to this aspect of research and holds considerable relevance for surgeons involved in the perioperative management of DM patients with GC.

## 5. Conclusion

Malignant tumors are a leading cause of death among patients with DM. Although the exact etiology of DM with concurrent GC remains unclear, current theories suggest a potential association with autoimmune and genetic factors. There is a possibility that DM may act as a precursor to the development of gastric cancer, and recognizing this association may enable earlier diagnosis and improved cancer treatment outcomes. Surgical management of DM patients with concurrent gastric cancer poses increased risks of complications and prolonged hospitalization. Surgeons should approach these cases with heightened caution. Further investigations are necessary to elucidate the underlying pathophysiological mechanisms contributing to the increased incidence of complications, with the goal of enhancing the prognosis of patients with DM and concurrent gastric cancer.

## Data Availability

The original contributions presented in the study are included in the article/Supplementary Material, further inquiries can be directed to the corresponding author.
